# Prospective clinical feasibility assessment of the Osteoporotic Pelvis Fracture Score (OFP-Score)—a multicenter prospective cohort study

**DOI:** 10.1186/s12877-026-07682-6

**Published:** 2026-05-23

**Authors:** Ulrich J. A. Spiegl, Pamela Schanderl, Volker Zimmermann, Bernhard Ullrich, Martin Naisan, Felix Kohler, Erik Wegner, Michael Müller, Frank Hartmann, Klaus J. Schnake, Imke Schmerwitz, Lars Behr, Max J. Scheyerer, Robert Pätzold, Sebastian Grüninger, Thomas Mendel, Georg Osterhoff, Working Group Osteoporotic Fractures of the Spine Section of the German Society of Orthopaedics and Trauma

**Affiliations:** 1 Department of Orthopaedics, Trauma and Plastic Surgery, University Hospital Leipzig, Leipzig, Germany; 2 Clinic for Trauma Surgery and Orthopaedics, Munich Harlaching, Sanatoriumspl. 2, München, 81545 Germany; 3 Department of Trauma and Orthopedic Surgery, Klinikum Traunstein, Traunstein, Germany; 4 Department of Trauma and Reconstructive Surgery, BG Klinikum Bergmannstrost Halle, Halle, Germany; 5 Department of Trauma and Orthopedic Surgery, St. Josef-Hospital Wiesbaden, Wiesbaden, Germany; 6 Department of Trauma, Hand and Reconstructive Surgery, Universitätsklinikum Jena – Friedrich-Schiller-University, Jena, Germany; 7 Department of Trauma and Orthopedic Surgery, Universitätsmedizin Mainz – Johannes Gutenberg-University, Mainz, Germany; 8 Department of Orthopedic and Trauma Surgery, Universitätsklinikum Schleswig-Holstein, Campus Kiel, Germany; 9 Department of Trauma Surgery, Diakonie Krankenhaus, Bad Kreuznach, Germany; 10 Center for Spinal and Scoliosis Surgery, Malteser Waldkrankenhaus St. Marien, Erlangen, Germany; 11 Clinic for Orthopedic Surgery, Sports Traumatology and Trauma Surgery, Städtisches Klinikum Wolfenbüttel, Wolfenbüttel, Germany; 12 Interdisciplinary Center for Spine and Neurotrauma, Sana Klinikum Borna, Borna, Germany; 13 Department of Trauma and Orthopedic Surgery, Universitätsklinikum Düsseldorf – Heinrich Heine University, Düsseldorf, Germany; 14 Department of Trauma and Reconstructive Surgery, BG Unfallklinik Murnau, Murnau, Germany; 15 Department of Orthopedics and Traumatology, Klinikum Nürnberg, Nuremberg, Germany

**Keywords:** Osteoporosis, Pelvic ring fracture, Operative therapy, Conservative therapy, OF Pelvis score, Treatment decision

## Abstract

**Purpose:**

To evaluate the feasibility of using the recently developed OF-Pelvis-Score (OFP-Score) for treatment decisions in patients with osteoporotic fractures of the pelvis (OFP) based on standard clinical diagnostics.

**Methods:**

A multicenter prospective cohort study was conducted at 14 trauma centers including 375 consecutive patients who were treated for an OFP over a period of 19 months. All fractures were classified according to the OF-Pelvis-Classification (OFP-Classification). The decision for either conservative or surgical therapy was made independently of the OFP-Score recommendation. Final decisions were compared to the recommendations given by the OFP-Score.

**Results:**

375 patients with an average age of 81.0 years (± 7.6) were included, mainly female (85.6%). According to the OFP-Score, surgery was recommended in 60.5%, and conservative treatment was recommended in 21.9%. In 66 patients (17.6%) the score was undetermined with no treatment recommendation. In daily practice, 33.6% of the patients were treated conservatively and the remaining 66.4% operatively. Overall, the agreement between the OFP-Score and the performed treatment was 91%. The score was obtained in a mean of 3.1 min (± 2.9). All patients improved significantly with respect of VAS (*p* < 0.001) and ODI (*p* < 0.001) during their hospital stay.

**Conclusion:**

Patients with osteoporotic sacral fractures improved clinically both after surgical and conservative treatment. The OFP-Score-based therapy recommendations showed a promisingly high rate of agreement with the therapy of daily practice. The scoring method provides a structured framework that supports clinical decision-making, complementing clinical judgment and other evidence-based tools in guiding treatment choices.

## Introduction

The incidence of osteoporotic fractures of the pelvis (OFP) has significantly increased over the past few years [1]. OFP are a unique entity and the clinical picture varies, ranging from isolated fracture edema to complex transiliac or transiliosacral fractures [2]. The mechanism of trauma, the clinical picture and the goals of treatment in patients with OFP are very different from those observed in young individuals with a higher level of activity, stronger bone and less comorbidities [3, 4]. OFP are usually isolated injuries as a result of a minor trauma [5]. More than 80% of the patients with OFP have at least two systemic diseases in their past medical history [5]. Pre-existing disability, gait abnormalities and impaired bone quality are common in this population and can be risk factors for future fractures and thus influence the outcome [6–8]. For the majority of OFP non-operative treatment is possible. However, in contrast to younger patients, patients with OFP need to be mobilized as early as possible to avoid the characteristic complications of the immobilized elderly. The standard systems for classification (Tile, OTA/AO, Young & Burgess, Denis) have been used for high energy osteo-ligamenteous injuries of the pelvic ring [9–11]. Their purpose is to predict instability, need for transfusion and the presence of concomitant injuries. This does not resemble the injury pattern found in pelvic fragility fractures after a low-energy trauma, where instability has to be defined differently [2, 12].

Hence, Rommens and Hofmann have proposed the FFP classification, a purely morphological CT-based system that has become widely accepted for categorizing these fractures [2]. However, the fracture morphology alone does not allow for a definitive conclusion regarding whether an operative or conservative treatment strategy should be pursued.

**Fig. 1:**
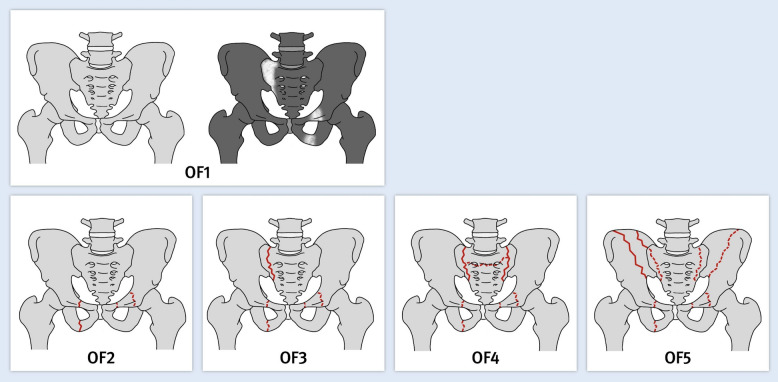
The OFP-Classification is presented: OF1: edema in the pelvic ring at one or more locations (no evidence of fractures on CT); OF2: anterior fracture of the pelvic ring on one or both sides; OF3: unilateral sacral fracture with or without fracture of the anterior pelvic ring; OF4: bilateral sacral fractures with or without fracture of the anterior pelvic ring including H- or U-type fractures; OF5: iliac or transiliosacral fracture with or without fracture of the anterior pelvic ring. Solid lines are variants for the fractures of the different OFP fracture types: dashed lines are optional additional fracture localizations. MRI or dual energy CT is required for edema detection

The OF Pelvis classification, a novel classification system for osteoporotic fractures, developed by the Working Group “Osteoporotic Fractures” (AG-OF) of the Spine Section within the DGOU, ensures reliability and reproducibility for clinical application (Fig. 1) [13]. In a next step, a score for therapeutic decision making based on the OFP-Classification was developed (Table 1) [14]. The aim of this study was to evaluate the feasibility of using the recently developed OFP-Score for treatment decisions in patients with OFP based on standard clinical diagnostics. In addition, the study sought to assess surgeons’ adherence to the scores recommendations.

**Table 1:** OF-pelvis score

Feature	Severity	Points
Fracture Morphology	OF-Pelvis Classification 1–5 (× 2)	2—10
Mobilization	Immobilized; severely restricted/ previously immobilized; sufficiently mobile	2; 0; −2
Pain (VAS)	≥ 5; < 5	1; −1
Neurology	Fracture-related neurological deficit present	1
Health Status	ASA > 3; mFI ≥ 2; Anticoagulants: Yes	Each −1; max −2
Modifiers	M1 = L5 transverse fracture on CT M2 = Fracture dislocation M3 = Fracture edema at an additional location (MRI)	Each 1, max 1

If a feature cannot be assessed or is unknown, 0 points are assigned. 0–7 points: Recommendation for conservative therapy. 8 points: Relative indication for surgery. 9–15 points: Recommendation for operative therapy

*ASA* ASA Score, *mFI* modified Frailty Index, *CT* Computed tomography, *MRI* Magnetic resonance imaging

## Materials and methods

A multicenter prospective cohort study was conducted at 14 trauma centers in Germany as an internal feasibility assessment. The study was approved by the local institutional ethics committees of each participating center. All patients gave written informed consent. The study aimed to evaluate a recently established classification of osteoporotic fractures (OFP-Classification) and an associated score that can be used to support treatment decisions (OFP-Score, Table 1) [14]. The OFP-Score was developed by the working group “Osteoporotic Fractures” of the Spine Section and members of the Pelvic Section of the German Society of Orthopaedics and Trauma. Several study centers that participated in this study have been involved developing and introducing the OFP-Score. A score of more than 8 points indicates recommendation for surgical treatment.

### Patients

In this study, 375 consecutive patients aged over 60 years who were treated operatively or non-operatively in one of the participating centers for an isolated OFP between May 2022 and December 2023 were included. Patients with high energy pelvis fractures, with metastatic disease or primary bone tumors of the pelvis, and patients with concomitant injuries were excluded.

All patients underwent the diagnostic work-up as per the standard protocols of the respective study center. This included in all centers: a clinical history and physical examination including assessment for neurological deficits, conventional AP radiographs of the pelvis and either a computed tomography scan (CT) or magnetic-resonance imaging (MRI) of the pelvis or both; additional diagnostics were performed if deemed necessary by the treatment teams. Fractures were classified according to the OFP-Classification [13].

The patients’ physical status was assessed by the ASA Physical Status Classification System. To assess frailty as an important parameter for evaluating health status as a risk factor, the modified 5-Item Frailty Index (mFI-5) was used upon admission [15]. This score consists of 5 items and a value greater than 2 is considered a risk factor for perioperative complications. The degree of mobilization was categorized into immobile, non-independently mobile, and independently mobile, where independently mobile is defined as the ability to move without assistance but with the use of aids (e.g., walking stick, crutches, walker) at the ward level (> 50 m).

The OFP-Score (Table 1) was applied and recorded but treatment was made as decided by the surgeon on call [14]. If the treatment decision deviated from the OFP-Score’s recommendation, reasons were documented. The time needed for obtaining the score was documented. Additionally, the satisfaction of the score in daily practice for each case was recorded ranging from 1 (very good) to 6 (unsatisfactory).

### Data acquisition and outcome measurement

At the time of admission, the day of treatment decision, and the day of discharge the following instruments for outcome measurements were assessed: 1. Visual Analogue Scale (VAS) under adjusted analgesia (up to a maximum of WHO step 2) during mobilization. If mobilization was not possible, pain should be evaluated during position changes (such as repositioning in bed or during toilet activities), 2. the Oswestry Disability Questionnaire (ODI) ^10^, and the 3. the Timed Up & Go test (TUG).

The primary outcome was the functional outcome, assessed using the Oswestry Disability Index (ODI). Secondary outcome parameters included pain and the mobility obtained by the TUG-test.

#### Statistical analysis

All data was entered into a web-based RedCap database and exported to SPSS 29.0 (SPSS Inc., Chicago, IL, USA) for statistical analysis. Unless otherwise denoted, data was summarized as mean with standard deviation (SD) [16].

Differences between admission, day of treatment decision and discharge in the primary outcome parameters were analysed using pair-sample t-test for continuous data and Chi-Square tests for categorical data. Additionally, the patients treated surgically and conservatively were compared using two-sample t-test. The level of significance was defined as p < 0.05.

## Results

The patient characteristics are depicted in Table 2. The patients’ average age was 81 years ranging from 60 to 99 years. Most patients were female. Low energy accidents like a fall from standing height were most frequent (71%). No trauma could be recalled in 17.7% of the patients. Most fractures were classified as type 3 or 4 fractures of the OFP-Classification. The most common modifier was fracture dislocation that was recorded in 29.1% of the cases. Bone edema of the pelvis in addition to the visible fracture shown in CT was found in 14.9% of the patients in the MRI. On admission, 55.2% of the patients could not be mobilized. The day of treatment decision was on average 3.4 days after admission. The average OFP-Score was 9.0 and 33.6% of the patients were treated conservatively. The average time for obtaining the OFP-Score was 3.1 min and the satisfaction using the score in daily practice was 1.4. The surgical strategies that were used are shown in Table 3.

**Table 2:** Baseline data

**Included patients**	**375**	
Age	81.0 ± 7.6 Years	
ASA	1 2 3 4 Missing	3 (1.6%) 141 (37.6%) 205 (54.7%) 14 (3.7%) 9 (2.4%)
Gender	85.6% female	
OF-Pelvis Classification	OF 1 OF2 OF3 OF4 OF5 Modifier 1 Modifier 2 Modifier 3	3 (0.8%) 27 (7.2%) 168 (44.8%) 158 (42.1%) 19 (5.1%) 13.6% 29.1% 14.9%
FFP-Classification	1a 1b 2a 2b 2c 3a 3b 3c 4a 4b 4c	16 (7.0%) 2 (0.5%) 27 (7.3%) 70 (18.7%) 53 (14.2%) 15 (4.1%) 8 (2.2%) 33 (9.0%) 10 (2.7%) 122 (32.5%) 7 (1.9%)
Conservative Therapy:	126 (33.6 %)	
Inpatient stay [days]	12.6 ± 7.6	
OF-Score	9.0 ± 2.4	
Time obtaining the Score [min]	3.1 ± 2.9	

*ASA* American Society of Anesthesiologists (ASA) score, *FFP-Classification* Fragility Fracture of the Pelvis -Classification [2]; min: minutes

**Table 3:** Surgical therapies

Transiliosacral screw	176 (47.0%)
- One sided	76 (20.3%)
- Two sided	100 (26.7%)
Transsacral screw	59 (15.7%)
Lumbopelvic fixation	33 (8.8%)
Posterior horizontal fixation	10 (2.7%)
External fixation	1 (0.3%)
Anterior osteosynthesis	24 (6.4%)
- Anterior plate	13 (3.5%)
- Screw	11 (2.9%)

The conformity of treatment that was performed with the OFP-Score recommendation was 91.2%. A score higher than 8 points suggesting surgical treatment was seen in 61.5% of the patients. Surgical treatment was performed in 96.4% of these cases. The OFP-Score was below 8 points suggesting conservative treatment in 21.9%. Despite this, surgery was performed in 19.0% of the patients with a score below 8 points. The score was exactly 8 in 66 patients (17.6%) suggesting a relative indication for surgery. Operative therapy was performed in 39 (59.1%) of these patients, whereas conservative therapy was done in 27 patients (40.9%). There were no statistically significant differences between patients treated operatively versus non-operatively in respect of age (81.6 vs 82.3 years; *p* = 0.65), gender (74.4% female vs 88.1% female; *p* = 0.15), OFP-Classification (3.2 vs 3.3; *p* = 0.30), pain on the day of decision making (6.5 vs 6.0; *p* = 0.21), and mobility of the day of decision making (4.6 vs 4.2; *p* = 0.15), and at discharge (3.7 vs 4.0; *p* = 0.17). Patients treated operatively had a significantly longer duration of hospital stay (14.9 vs 9.5 days; *p* = 0.001) and significant lower pain level at discharge without being clinically important (3.8 vs 4.7; *p* = 0.031).

Generally, surgically and conservatively treated patients benefited from therapy and improved significantly between hospital admission and discharge for both ODI (admission: 74.9 ± 20.2; discharge: 53.8 ± 17.1; p < 0.001) and VAS (admission: 7.8 ± 1.6; discharge: 3.9 ± 1.6; *p* < 0.001). The percentage of all patients who were bedridden could be decreased from 55% to 2.4% during the hospital stay.

Patient characteristics and outcome parameters in dependence on the treatment strategy are shown in Table 4. Patients treated operatively had significantly higher OFP-Scores and a significantly longer inpatient stay. Patients treated conservatively had significant lower pain levels at the day of treatment decision and on discharge.

**Table 4:** Patients’ Characteristics and Outcome Parameters in Dependence on the Therapy Strategy

	**Conservative therapy**	**Operative therapy**	***p*-value**
Age	81.6 ± 8.2	80.6 ± 7.3	0.298
ASA Score	2.6 ± 0.6	2.6 ± 0.6	0.709
OF-Score	6.8 ± 2.2	10.1 ± 1.6	** < 0.001**
Inpatient stay [days]	9.6 ± 6.5	14.2 ± 7.7	** < 0.001**
VAS-Score (Decis)	5.6 ± 1.9	7.1 ± 1.5	** < 0.001**
Time-up-go-test (Decis)[s]	34.2 ± 21.9	37.3 ± 33.3	0.533
VAS-Score (dism)	4.1 ± 1.6	3.7 ± 1.5	**0.018**
ODI (dism)	55.6 ± 16.7	52.8 ± 17.2	0.147
Timed Up and Go Test (dism)	29.5 ± 18.0	27.4 ± 16.7	0.344

*Decis* Day of treatment decision, *S* Seconds, *Dism* Dismission

No statistically significant differences were seen in the TUG test and the ODI on the day of discharge between both treatment groups.

Patients treated in accordance to the OFP-Score recommendations were associated with significant lower pain scores at discharge (mean VAS: 3.8) compared to those who were not treated in accordance to the score (mean VAS: 4.5; *p* = 0.046). No statistically significant differences could be seen with respect to the ODI and TUG.

## Discussion

The most important findings of this study are the very high agreement rates between the therapy recommendations based on the OFP-Score and the therapy that was carried out in clinical practice, as well as the short period of time required to collect the score. Interestingly, the treatment in accordance with the score was associated with significantly lower pain levels at discharge even though this difference did not reach the level of clinical importance. Thereby, it has to be kept in mind that several centers that participated in this study have been involved in introducing the score. A score-independent decision making was aimed for. However, the OFP-Score might still influence the decision making.

Notwithstanding, the score appears to be a very useful parameter for everyday clinical practice. This means that even less experienced orthopaedic surgeons can provide an assessment of further treatment. Patients who receive conservative treatment can be treated on site without the need for a costly transfer to another hospital, while those who benefit from surgical treatment can be transferred early if this cannot be offered locally. The fact that it only takes a few minutes to collect the score makes it appear to be a simple score that can be collected quickly at any time. In addition, user satisfaction collecting the score in daily practice was graded as very good. Additionally, the patients benefited from therapy, both conservative and surgical treatment. Surgical treatment was associated with lower pain at discharge but a longer duration of in hospital stay. Patients treated in accordance with the OFP-Score were associated with lower pain levels at discharge, although this finding should be interpreted cautiously given the observational design. Hereby, differences in VAS pain scores were small without being clinically important.

Overall, there is a consensus that an isolated consideration of fracture morphology is not sufficient to make an adequate treatment decision in challenging geriatric patients with pelvic insufficiency fractures [12, 14, 17]. Nevertheless, the fracture can provide information about fracture stability. The first-class analysis of fractures integrated into the FFP classification has made a very important contribution here [2]. Nevertheless, the FFP-classification is based on the high number of subtypes a challenge for the less experienced orthopedic surgeon potentially leading to misclassification or insufficient classification rates due to time constraints. In addition, the increasing influence of MRI imaging is not taken into account in the classification. The need for an MRI is stated in literature and can be seen by the quite high rate of positive third modifier [18]. A bony edema in a location that was not detected in CT was visible in about every seventh patient. This can change the therapy concept in surgically treated patients. Implementation of either transiliosacral screws on both sides or using transsacral screws are necessary in patients with a contralateral sacral bony edema whereas one sided iliosacral screws are possible in those with unilateral sacral fracture [17]. By including the MRI into the classification and due to simplicity leading to a high inter- and intra-rater reliability the OFP-Classification seems to be classification tool that might be used even more widely in everyday practice or can be a suitable supplement in addition to the FFP classification [13].

In general, in addition to the fracture morphology, the decisive factors that are taken into account in the treatment decision are the pain situation and the patient's ability to mobilize [12, 19]. Additionally, risk factors that can trigger surgical complications are taken into account. As a rule, the decision is made individually by an experienced surgeon. The OFP-Score integrates all the decision criteria mentioned above. The retrospective survey of 107 patients showed a similar agreement of 94% between the treatment recommendation of the OFP-Score and the treatment actually carried out [14]. This high agreement rate was confirmed by this prospective multicenter study. Thus, the OFP-Score seems to be a promising decision making tool transforming it to a more comprehensible process.

The applied treatment led to significant lower pain levels and higher mobility levels. Hereby, the pain level after surgical treatment was significantly lower at discharge compared to patients treated conservatively. This can be partly explained by the significant longer inpatient stay leading to further pain reduction. Generally, pain levels in both patient groups were comparable at admission. Thus, the significant higher OFP-Score in surgically treated patients can be explained by more complex fracture morphologies resulting in higher OFP-Classification types and less clinical improvement after initiating adequate analgesic medication. Patients with a OFP-Score of 8 were treated in a similar proportion nonoperatively and surgically with similar results during the hospital stay. These patients benefited similarly from both treatment strategies. Thus, more time should be taken for decision making. Those patients who further improve clinically will be ideal candidates for nonoperative treatment whereas those with persistent limitation and pain might benefit more from surgery.

The heterogeneity of surgical treatment for sacral insufficiency fractures is in accordance with the literature and can be explained by the high number of centers that participated in the study and the low level of evidence for each treatment strategy [17, 18, 20, 21]. However, in most patients transiliosacral or transsacral screws were used for the surgical treatment of sacral insufficiency fractures and only in minorities lumbopelvic fixation or horizontal fixators were implemented. Additionally, the number of patients treated anteriorly was low. Thus, the different therapy strategies could not be compared sufficiently.

Nevertheless, this study has some limitations that need to be mentioned. First of all, complications were not analyzed in this study. An interpretation of all complications is very important for decision making. However, these interpretations need to be done in detail elongating the manuscript tremendously. Thus, this will be done in a separate study aiming for the analysis of all complications.

Generally, the limitation section will be divided first into those that are related to the study design and second into those that are related to interpretational concerns. As mentioned before, the study was conducted in centers involved in the development of the OFP-Score. This can play a role in the treatment decision, meaning that a selection bias cannot be ruled out here. In the future the OFP-Score needs to be re-evaluated by a separate study group. An independent, external validation study with a minimum of 6-month follow-up is the next required step before this score can be endorsed for widespread clinical use. Furthermore, only the inpatient course was followed, meaning that it cannot be proven whether the treatment strategy also proved to be positive for the patient in the further course. In addition, the study design cannot answer the question of whether the therapy initiated was actually the best therapy for the patient. The surgical treatment strategies in particular differ greatly and are only homogeneous to a limited extent among the participating clinics. This is due to the low level of evidence for individual strategies.

The authors acknowledge that the lack of external validation of our findings may limit the generalizability of the results, particularly to centers with varying levels of experience or different available resources. The applicability of our findings may be less certain in less experienced centers or those with different clinical infrastructures, where treatment protocols and patient management approaches may differ. Therefore, caution is advised when interpreting the results in such settings, and further studies involving external validation and a long- term follow-up are needed to confirm the applicability of our findings across a broader range of clinical environments. Additionally, the statistically significant differences in pain level between patients that were treated in accordance with the OFP-Score and those that were not treated in accordance with it were not clinically significant.

The study’s aim was to evaluate the effectiveness of the treatment from the patient's perspective, which is why complications were not analyzed in detail. Again, this is a relevant weakness of this study. However, we recognize the importance of addressing safety concerns in future studies, and we suggest that incorporating a more comprehensive analysis of complications would complement the assessment of patient-reported outcomes and provide a more complete picture of treatment effectiveness.

## Conclusion

Patients with osteoporotic sacral fractures improved clinically both after surgical and conservative treatment. The OFP-Score-based therapy recommendations showed a promisingly high rate of agreement with the therapy of daily practice. The scoring method provides a structured framework that supports clinical decision-making, complementing clinical judgment and other evidence-based tools in guiding treatment choices. Future studies need to re-evaluate the OFP-Scores including a detailed complication analysis.

## Data Availability

All relevant raw data, will be freely available to any scientist wishing to use them for non-commercial purposes: Please contact: uli.spiegl@gmx.de.
